# Supplementary figures and images for: Yellow sea mediated segregation between North East Asian Dryophytes species
Source: PLoS One. 2020 Jun 24;15(6):e0234299. doi: 10.1371/journal.pone.0234299 (PMC7314424; doi:10.1371/journal.pone.0234299)

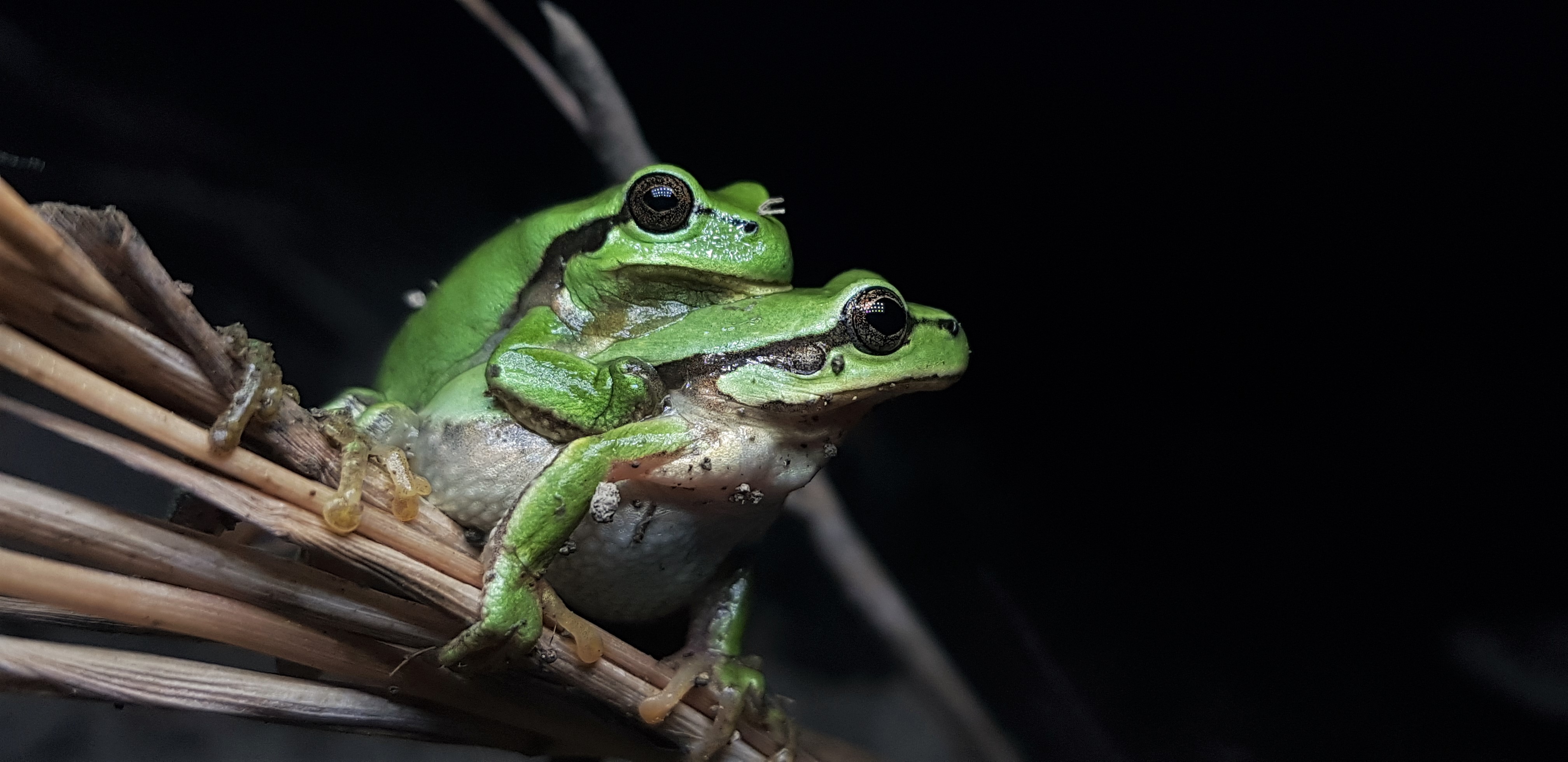

Supplement: S1 Fig — (JPG) [file pone.0234299.s002.jpg]
