# Supplementary material for: Yellow sea mediated segregation between North East Asian Dryophytes species
Source: PLoS One. 2020 Jun 24;15(6):e0234299. doi: 10.1371/journal.pone.0234299 (PMC7314424; doi:10.1371/journal.pone.0234299)
Supplement: Attachment — Submitted filename: Answer to reviewer.docx [file pone.0234299.s004.docx]

Dear Dr. Borzée and Dr. Min,

I am glad to read the revised version and found that most comments from the previous reviewers have been revised. In order to facilitate the timing of publication, your manuscript does not need another round of review by the original referees. Nevertheless, there are a few minor problems which need to be revised before the manuscript could be accepted. I would be more than happy to see the paper to be published as soon as possible if these editing problems could be solved.

**=> Dear Editor, thank you very much for the support, we have now resubmitted the corrected version online.**

Fig. 1. In the previous comments, we have proposed the redundancy of the outline of Anhui Province. After consideration, I still felt it redundant and even misleading. Hefei and Chuzhou appear at the upper part of Anhui Province, whereas the label of “Anhui” appears at the lower part, and this makes the readers confused. In this case, the precise position of the province is not important information for the readers. I suggest to eliminate the outline, and label the two localities as “Hefei, Anhui Prov.” and “Chuzhou, Anhui Prov.” This solves most the problems.

**=> We have modified the text on the figure following your recommendation, omitting the abbreviation “prov.”. The text now reads “Hefei (Anhui)”.**

Fig. 1. As proposed by our previous comment, some colors used in the figure were similar. The major problem raised from colors of D. suweonensis and D. flaviventris; these two are not easily distinguished by color-blinded people. Therefore, please change one of them.
**==> We have modified the range of D flaviventris, it is now orange, and all figures with colour coding have been updated accordingly.**

Fig. 2. I think you submitted the old version; please update. The photos, the sonograms, and the labels are still overlapping with the STRUCTURE probabilities.

**=> The new figure is indeed very similar, only the height of some barplots and the values of the delta K changed following the additional analyses. The sonograms are now removed from the newly submitted figure, but we maintained labels and pictures on the graph as it is not overlapping with the sections of the bar plots that are showing multiple clade assignment.**

Materials and methods, lines 103 – 104:
I suspected that you have misunderstood the meaning of the reviewer. There was a missing link about how you identify, and how you assign your samples to these three clades. Therefore, the reviewer was asking about the definition of “the three clades”, not the definition of “what a clade means”. I think you need a few sentences to clarify how you distinguish, how you identify, and how you assign the samples to the three clades. By sample locations? Morphology? Or some other pre-tests?
**=> The individuals were assigned based on sample location, until genetic analyses, upon which samples from the Korean Peninsula were re-assigned to either of the two clades based on the results of the genetic analyses. This information was added to the manuscript such as: “Call properties and morphological differences between clades were not known before this study, and therefore individual sampled in China were assigned to *D. immaculatus*, based on range, and individuals sampled in R Korea and DPR Korea were assigned to either *D. suweonensis* or the new clade based on the genetic analyses”.**

Minor revisions
Line 89: lack of a full stop at the end of the sentence.

**=> Corrected as suggested**

Line 1242: “includes”

**=> Corrected as suggested**
